# Efficacy and Safety of Vibegron Add‐On Therapy for Persistent Overactive Bladder Symptoms in Benign Prostatic Hyperplasia Patients With α_1_‐Blocker Treatment: A Multi‐Center Prospective Randomized Controlled Study (VATON Study)

**DOI:** 10.1111/luts.70053

**Published:** 2026-03-13

**Authors:** Masaki Yoshida, Yoshinori Nishino, Hiroshi Nagae, Shinobu Kato, Sadaaki Sakamoto, Morifumi Hojo, Toshihide Miyauchi, Shinji Kageyama, Shinichi Takahashi, Hideya Kuroda, Naoki Mori, Yasuhiro Kasagi, Koichi Masunaga, Yoshiyuki Nabeshima, Masanori Nomiya, Koichi Iwashita, Koichi Miyamae, Masayuki Otani, Kazuya Kawahara, Makoto Ikeda, Shinichi Kubono, Nobuhiro Haga

**Affiliations:** ^1^ Department of Urology Sakurajyuji Hospital Kumamoto Japan; ^2^ Nishino Clinic Gifu Japan; ^3^ Nagae Prostate Care Clinic Shizuoka Japan; ^4^ Kato Urological Clinic Kanagawa Japan; ^5^ Department of Urology Nakamura Hospital Oita Japan; ^6^ Department of Urology Koga Hospital 21 Fukuoka Japan; ^7^ Department of Urology Oita Urology Hospital Oita Japan; ^8^ Kageyama Clinic Shizuoka Japan; ^9^ Takahashi Urological Clinic Oita Japan; ^10^ KURODA UROLOGY CLINIC Osaka Japan; ^11^ Department of Urology Tagawa Municipal Hospital Fukuoka Japan; ^12^ Kasagi Urology Clinic Oita Japan; ^13^ Itabashi Tokumaru Clinic Tokyo Japan; ^14^ Department of Urology Fukuoka Tokushukai Hospital Fukuoka Japan; ^15^ Department of Urology National Center for Geriatrics and Gerontology Aichi Japan; ^16^ Shiizako Urological Clinic Oita Japan; ^17^ Maehara Urology Clinic Kumamoto Japan; ^18^ Otani Clinic Oita Japan; ^19^ KAWAHARA Nephro‐Urology Clinic Kagoshima Japan; ^20^ Medical Affairs, Kyorin Pharmaceutical Co. Ltd. Tokyo Japan; ^21^ Medical Department Kissei Pharmaceutical Co. Ltd. Tokyo Japan; ^22^ Department of Urology Fukuoka University, Faculty of Medicine Fukuoka Japan

**Keywords:** adrenergic alpha‐1 receptor antagonist, overactive bladder, prostatic hyperplasia, randomized controlled trial, vibegron, *β*_3_‐adrenergic receptor agonist

## Abstract

**Objectives:**

To evaluate the efficacy and safety of vibegron add‐on therapy for persistent overactive bladder (OAB) symptoms after *α*_1_‐blocker monotherapy for benign prostatic hyperplasia (BPH).

**Methods:**

Eligible patients were aged ≥ 50 years and diagnosed with BPH. All patients had received an *α*_1_‐blocker for ≥ 8 weeks, yet had persistent OAB symptoms. Patients were randomized 1:1 to receive add‐on vibegron (50 mg) or to continue *α*_1_‐blocker monotherapy. The primary efficacy endpoint was the between‐group difference from baseline (week 0) to week 12 in total overactive bladder symptom score (OABSS). Secondary endpoints included bladder diary parameters, OABSS subscores; International Prostate Symptom Score total, storage/voiding, quality‐of‐life score; and patient satisfaction assessed by the Patient Global Impression. Safety was assessed by recording treatment‐emergent adverse events.

**Results:**

Overall, 158 patients were randomized into two groups (*n* = 79 each). The least‐squares mean change in the primary endpoint (OABSS total score) was −1.9 (95% confidence interval [CI]: −2.4 to −1.5) with *α*_1_‐blocker monotherapy and −3.3 (95% CI: −3.8 to −2.9) with vibegron add‐on therapy; the between‐group difference was −1.4 (95% CI: −2.0 to −0.8; *p* < 0.001), indicating a significant improvement with add‐on therapy. Across the secondary endpoints, favorable outcomes were observed. Higher satisfaction was reported in the vibegron add‐on therapy group than in the *α*_1_‐blocker monotherapy group. Vibegron was well tolerated, and no serious drug‐related treatment‐emergent adverse events were observed.

**Conclusions:**

Vibegron add‐on therapy to an *α*_1_‐blocker may be effective and safe for treating BPH with persistent OAB symptoms.

## 1 Introduction

Benign prostatic hyperplasia (BPH) is a progressive disease frequently observed in middle‐aged and older men. Prostatic enlargement can cause both voiding dysfunction and various storage symptoms, including those associated with overactive bladder (OAB), thereby significantly affecting quality of life (QOL) [1, 2]. Therefore, effective pharmacological interventions are essential for patient management. The first‐line pharmacological treatment for BPH is *α*_1_‐adrenoceptor antagonists (*α*_1_‐blockers), with representative agents including tamsulosin, naftopidil, and silodosin. According to the clinical guidelines for male lower urinary tract symptoms and BPH, all of these agents are assigned a Grade A recommendation [3]. Nevertheless, storage symptoms, including OAB, often persist after *α*_1_‐blocker monotherapy; hence, combination therapy with OAB agents such as anticholinergics is recommended in the clinical guidelines for overactive bladder syndrome [4].

Recently, growing concerns about anticholinergic adverse effects, including effects on cognitive function, have shifted prescribing practices from anticholinergic agents to *β*_3_‐adrenoceptor agonists [5]. Notably, the combination therapy of an *α*_1_‐blocker and the *β*_3_‐adrenoceptor agonist mirabegron for treating OAB symptoms in patients with BPH has been demonstrated to be effective and safe in large‐scale randomized controlled trials (RCTs) and is currently assigned a Grade A recommendation [6, 7].

Vibegron is a selective *β*_3_‐adrenoceptor agonist that has shown favorable efficacy and safety profiles in phase III clinical trials [8, 9]. It received the first regulatory approval worldwide in Japan in September 2018 for the treatment of urgency, increased daytime frequency, and urgency urinary incontinence associated with OAB. Nevertheless, additional evidence is warranted for patients with OAB accompanied by BPH, as the efficacy and safety of vibegron add‐on therapy with *α*_1_‐blockers in this population have been demonstrated only in small‐scale studies [10, 11].

Here, we conducted an RCT with a larger patient population to compare the efficacy and safety of add‐on vibegron therapy with that of continued *α*_1_‐blocker monotherapy in patients with BPH with persistent OAB symptoms despite ongoing *α*_1_‐blocker treatment.

## 2 Methods

### 2.1 Study Design and Patients

This study was a multicenter, open‐label, randomized, parallel‐group comparative study conducted at 20 institutions in Japan. The main entry criteria were as follows: men aged ≥ 50 years at the time of consent; a diagnosis of BPH by basic evaluation and other diagnostic methods; use of *α*_1_‐blocker (silodosin, tamsulosin, or naftopidil) at the same dosage for ≥ 8 weeks before Visit 1; written consent; prostate volume ≥ 20 mL; and an OAB symptom score (OABSS) Q3 of ≥ 2 (urgency episodes/week ≥ 1) with an OABSS total score of ≥ 3 [12]. All inclusion and exclusion criteria are listed in Table S1.

After providing written informed consent, patients were enrolled in an observation period during which they completed a 3‐day bladder diary. Subsequently, eligible patients were randomly assigned at baseline (week 0) to either the vibegron add‐on therapy group, which received vibegron 50 mg once daily in addition to an *α*_1_‐blocker, or the *α*_1_‐blocker monotherapy group, which continued *α*_1_‐blocker treatment without vibegron. Patients were randomized in a 1:1 ratio using a centralized registration system. A 12‐week observation period was then established. The minimization method was employed using adjustment factors, including the OABSS total score (≥ 8 or < 8) and the type of α_1_‐blocker (silodosin, tamsulosin, or naftopidil) used at Visit 1 (week 0).

During the 12‐week study period, OABSS was used at weeks 0, 2, 4, 8, and 12 to assess the OAB symptoms of patients. International prostate symptom score (IPSS)/IPSS‐QOL was evaluated at weeks 0, 4, 8, and 12. Bladder diaries were maintained for 3 days prior to weeks 0 and 12. The proportion of patients who achieved the minimal clinically important change (MCIC), defined as a decrease of ≥ 3 points in total OABSS score from baseline [13], was calculated. Patient satisfaction with treatment was evaluated at 12 weeks using the Patient Global Impression (PGI) [14], which employs a seven‐point rating scale ranging from 1 (very much improved) to 7 (very much worse), with intermediate scores representing much improved (2), minimally improved (3), no change (4), minimally worse (5), and much worse (6). PGI ratings of 1 and 2 were categorized as “very much satisfied”, while PGI ratings of 1, 2, and 3 were categorized as “satisfied”. Maximum urinary flow rate (Qmax) and post‐void residual urine volume (PVR) were evaluated at weeks 0 and 12. The study design is illustrated in Figure S1.

Concomitant medication use during the study was managed as follows. New BPH treatments, OAB medications, desmopressin, and bladder training were prohibited. Pre‐existing therapy with Kampo medicines for urological conditions, drugs with anticholinergic effects other than those used to treat OAB, *α*/*β*‐blockers, and *α*‐agonists was only permitted with stable doses.

### 2.2 Outcomes

The primary efficacy endpoint was the between‐group difference in the OABSS total score change from baseline (week 0) to week 12 between the vibegron add‐on therapy and the *α*_1_‐blocker monotherapy groups. Secondary endpoints included between‐group differences in mean bladder diary parameter changes from baseline (week 0) to week 12: micturition number per 24 h, voided volume per micturition, number of urgency episodes per 24 h, number of urgency urinary incontinence episodes per 24 h, nocturia episodes per night, hours of undisturbed sleep (HUS), Qmax, and PVR.

Additionally, OABSS, IPSS (total score, storage symptom score, and voiding symptom score), and IPSS‐QOL score were evaluated for between‐group differences in changes from baseline (week 0) at each time point. Other endpoints included between‐group differences in PGI. Subgroup analyses evaluated the change from baseline in the OABSS total score. Prespecified subgroups included age (< 75 and ≥ 75 years), prostate volume (< 50 and ≥ 50 mL), OAB duration (< 7 and ≥ 7 months), complications (no and yes), OABSS total score (< 8 and ≥ 8), and *α*_1_‐blocker type (silodosin, tamsulosin, and naftopidil). Safety evaluations included all adverse events that occurred during the study. Drug‐related treatment‐emergent adverse events (TEAEs) were evaluated in the vibegron add‐on treatment group.

### 2.3 Statistical Analyses

The sample size was calculated based on OABSS data changes from a previous RCT [15]. We assumed changes of −0.87 and −2.21 in the groups continuing *α*_1_‐blocker therapy and receiving additional vibegron, respectively, with a between‐group difference of 1.34 and a standard deviation of 3.0. To achieve 80% statistical power and a two‐sided *α*‐level of 0.05, a total sample size of 160 participants (80 per group) was required.

Efficacy analyses were conducted on the full analysis set, comprising all randomized participants excluding those with major protocol violations or consent withdrawal. Safety analyses were performed on the safety set, including all randomized participants except those who withdrew consent. A per‐protocol sensitivity analysis was conducted on protocol‐compliant participants (per‐protocol set).

For the primary endpoint, summary statistics for the OABSS total score at weeks 0 and 12 and the changes were calculated for each treatment group. The least squares mean changes, standard errors, and 95% confidence intervals (CIs) for each group were determined using the constrained longitudinal data analysis (cLDA) method. The cLDA model was performed using the SAS MIXED procedure. Additionally, the differences in least squares means between groups, SE, 95% CI, and *p*‐values for the tests were presented. Similar analyses were performed for secondary endpoints, including bladder diary parameters, Qmax, PVR, OABSS score, IPSS score, and IPSS‐QOL score.

For HUS, means or changes from baseline with 95% CI were presented, together with between‐group differences in means and their 95% CI. Regarding PGI, the populations of patients in each category with 95% CI were reported, with between‐group differences in proportions with 95% CI and P values from Fisher's exact test.

All statistical tests were two‐sided with a significance level of 5%, and all analyses were performed using the SAS software version 9.4 (SAS Institute, Cary, NC, USA). Adverse events were categorized using MedDRA/J version 28.0 (Medical Dictionary for Regulatory Activities, Japanese Edition).

### 2.4 Ethics Statement

This study was conducted in accordance with the ethical principles outlined in the Declaration of Helsinki and complied with the Japanese Clinical Trial Act [16]. The study protocol received approval from the Certified Review Board of Hattori Clinic Medical Corporation (jRCTs031230450) on November 17, 2023. All participants provided written informed consent prior to study enrollment.

## 3 Results

### 3.1 Patient Characteristics

**FIGURE 1:**
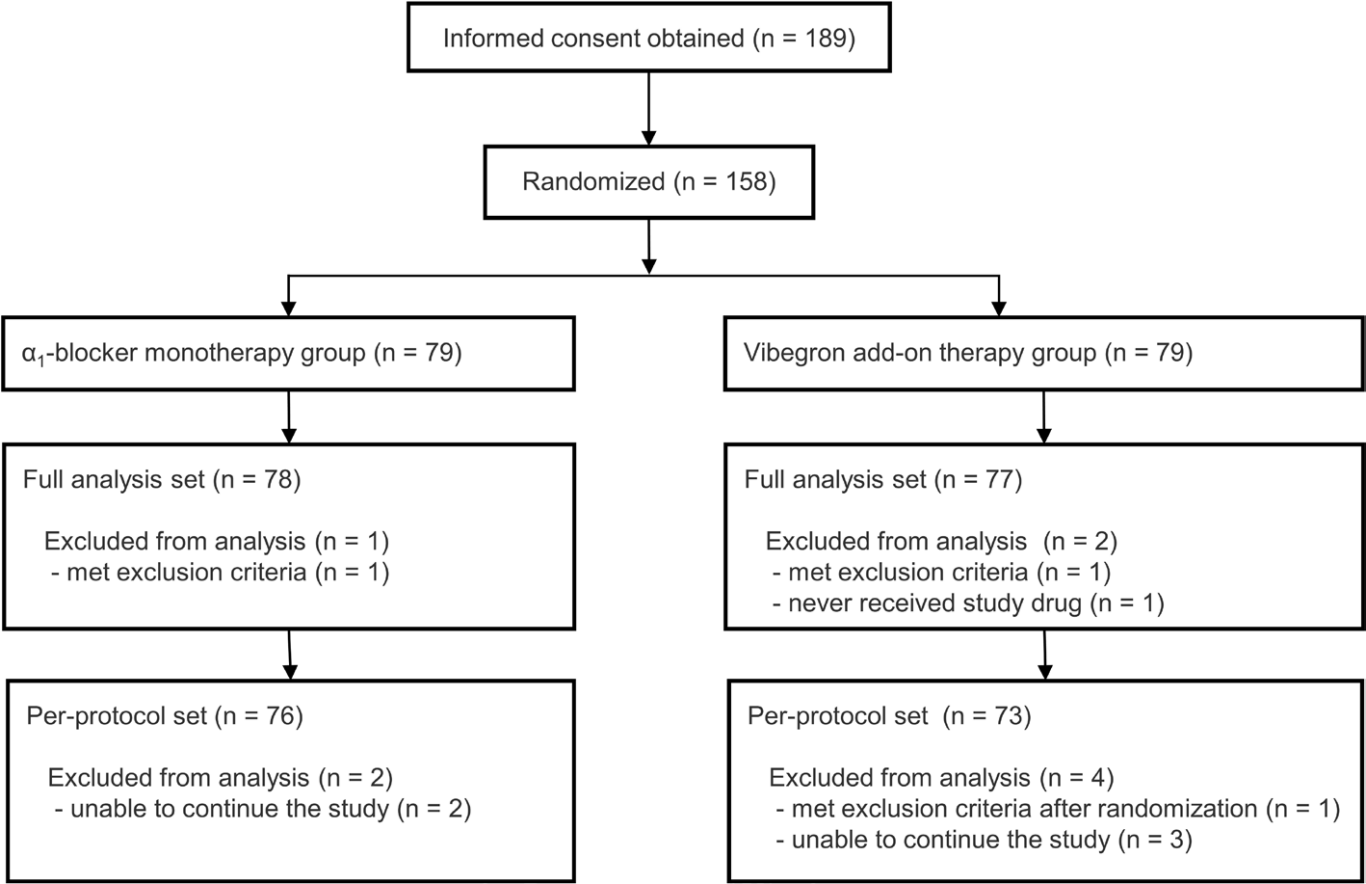
Flow diagram of participant inclusion.

This study was conducted between December 2023 and March 2025. Overall, 189 patients provided informed consent, and 158 patients met the eligibility criteria and were randomized to either the *α*_1_‐blocker monotherapy group (*n* = 79) or the vibegron add‐on therapy group (*n* = 79) (Figure 1). Of the 158 patients, 155 were included in the full analysis set. Patient demographic and baseline characteristics in the full analysis set population were comparable between the *α*_1_‐blocker monotherapy group and the vibegron add‐on therapy group (Table 1), with a mean (SD) age of 74.4 (6.4) and 73.9 (7.4) years, respectively. The distribution of baseline *α*_1_‐blocker medications was similar between the groups (*p* = 0.899). In the *α*_1_‐blocker monotherapy and vibegron add‐on therapy groups, the numbers of patients receiving silodosin, tamsulosin, and naftopidil were 23, 26, and 29, and 23, 23, and 31, respectively.

**TABLE 1:** Demographics and baseline clinical characteristics of the participants.

Parameter		*α*_1_‐blocker monotherapy group (*n* = 78)	Vibegron add‐on therapy group (*n* = 77)	*p*
Age (year), mean (SD)		74.4 (6.4)	73.9 (7.4)	0.8324
BMI (kg/m^2^), mean (SD)		23.5 (3.5)	24.1 (3.7)	0.5079
Prostate volume (mL), mean (SD)		36.8 (14.8)	37.1 (14.0)	0.9786
Duration of OAB (mo), mean (SD)		27.4 (55.8)	32.6 (44.8)	0.0548
OAB wet/dry		4/74	9/68	0.1595*
Complications, *n*	No	18	25	0.2127*
	Yes	60	52	
	Diabetes	13	8	—
	Hypertension	44	38	—
	Dyslipidemia	25	26	—
	Cardiovascular disease	13	14	—
	Constipation	7	6	—
*α*_1_ blocker, *n*	Silodosin	23	23	0.8990*
	Tamsulosin	26	23	
	Naftopidil	29	31	

*Note:* * indicates Fisher's exact test; all other comparisons used the Wilcoxon rank‐sum test.

Abbreviations: BMI, body mass index; CI, confidence interval; mo, months; OABSS, overactive bladder symptom score; Qmax, maximum urinary flow rate; SD, standard deviation.

### 3.2 Efficacy Endpoint

**FIGURE 2:**
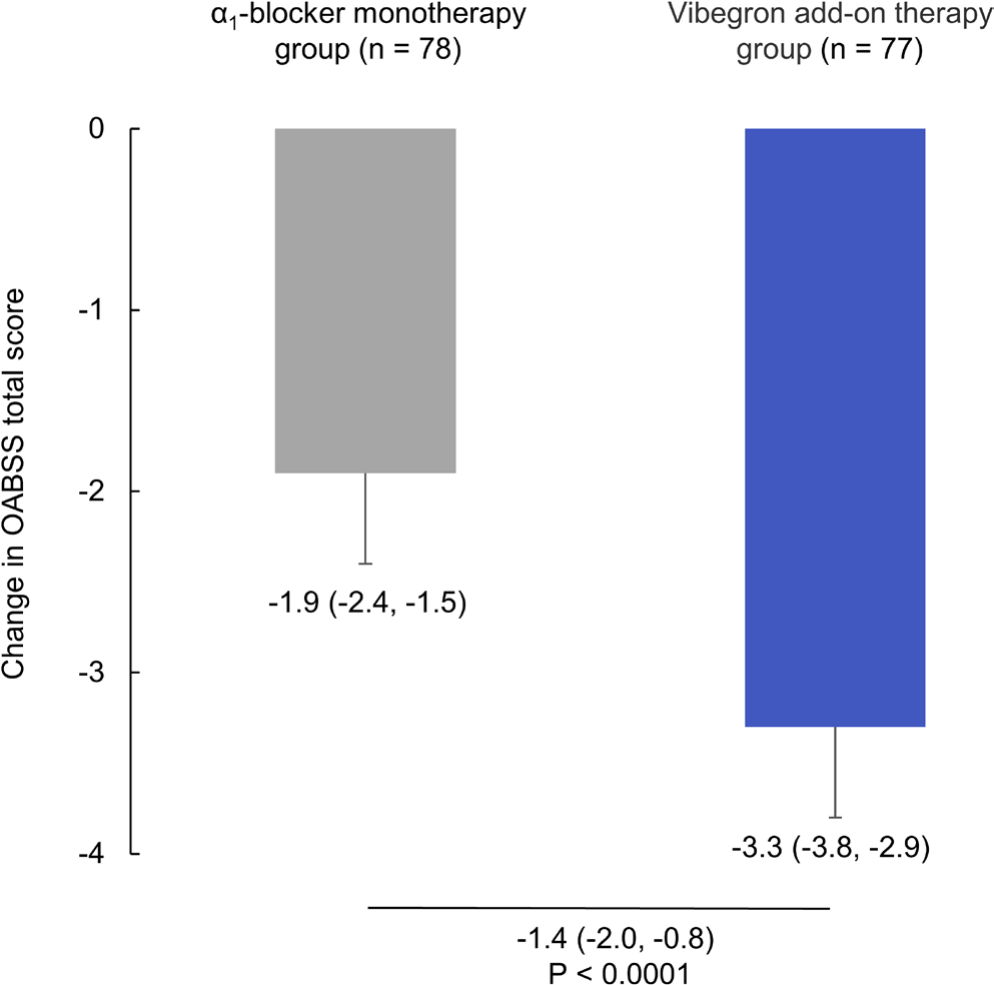
Change in OABSS total score from baseline to week 12. Values are shown as LS mean (95% CI). Boxes, bars and values below the bars indicate the LS mean (95% CI) and *p*‐value for the difference in change observed between groups. CI, confidence interval; LS, least squares; OABSS, overactive bladder symptom score.

The change in OABSS total score from baseline (week 0) to week 12, which was the primary endpoint, was −1.9 (95% CI: −2.4 to −1.5) and −3.3 (95% CI: −3.8 to −2.9) in the *α*_1_‐blocker monotherapy and vibegron add‐on therapy groups, respectively. The difference between groups was −1.4 (95% CI: −2.0 to −0.8, *p* < 0.001), demonstrating significant improvement in the vibegron add‐on therapy group compared to the *α*_1_‐blocker monotherapy group (Figure 2). In per‐protocol set analysis, the between‐group difference was −1.5 (95% CI: −2.1 to −0.9, *p* < 0.001).

The secondary endpoints were evaluated as follows: change in mean daily micturition frequency from baseline (week 0) to week 12 was −0.7 (95% CI: −1.2 to −0.3) and −1.7 (95% CI: −2.2 to −1.3) in the *α*_1_‐blocker monotherapy and vibegron add‐on therapy groups, respectively. The difference between groups was −1.0 (95% CI: −1.6 to −0.4; *p* < 0.001). Similarly, the vibegron add‐on therapy group demonstrated significant improvements in voided volume per micturition, urgency episodes, nocturia episodes, and HUS, but not in urgency incontinence episodes (Table 2).

**TABLE 2:** Efficacy results in the bladder diary variables, Qmax and PVR from baseline at week 12, and PGI value at week 12.

		*α*_1_‐blocker monotherapy group				Vibegron add‐on therapy group				Difference in changes between groups	
		*n*	Week 0	Week 12	Change (95% CI)	*n*	Week 0	Week 12	Change (95% CI)	Difference (95% CI)	*p*
Bladder diary	Micturitions/day	78	10.0 ± 2.7	9.2 ± 2.5	−0.7 (−1.2, −0.3)	77	10.1 ± 3.0	8.4 ± 2.5	−1.7 (−2.2, −1.3)	−1.0 (−1.6, −0.4)	0.0008
	Voided volume/micturition (mL)	78	168.7 ± 53.7	165.5 ± 54.9	−4.4 (−11.5, 2.8)	77	171.5 ± 46.2	198.8 ± 62.6	27.0 (19.8, 34.3)	31.4 (21.4, 41.4)	< 0.0001
	Urgency episodes/day	66	2.8 ± 2.2	1.7 ± 2.0	−1.0 (−1.6, −0.5)	65	2.9 ± 3.0	0.9 ± 1.8	−2.0 (−2.5, −1.5)	−1.0 (−1.7, −0.3)	0.0052
	Urgency urinary incontinence episodes/day	16	1.2 ± 1.4	0.5 ± 0.8	−0.4 (−1.1, 0.3)	16	1.2 ± 1.0	0.9 ± 2.3	−0.5 (−1.1, 0.2)	0.0 (−0.9, 0.8)	0.9160
	Nocturia episodes/day	77	1.9 ± 1.2	1.8 ± 1.0	−0.1 (−0.3, 0.1)	74	2.1 ± 1.1	1.6 ± 1.1	−0.5 (−0.7, −0.3)	−0.4 (−0.7, −0.2)	0.0014
	HUS (minutes)	78	216.2 ± 96.1	221.1 ± 98.5	5.7 (−15.1, 26.6)	77	199.1 ± 96.3	247.1 ± 123.0	44.4 (23.2, 65.5)	38.6 (10.7, 66.6)	0.0069
OABSS	MCIC responders, *n* (%)	76		36 (47.4)		75		50 (66.7)		19.3 (3.8, 34.8)	0.0214
PGI	Very much satisfied, *n* (%)	77		13 (16.9)		77		43 (55.8)		39.0 (25.1, 52.9)	< 0.0001
	Satisfied, *n* (%)	77		44 (57.1)		77		68 (88.3)		31.2 (18.0, 44.3)	< 0.0001
Qmax (mL/s)		78	12.0 ± 5.5	11.0 ± 5.1	−1.0 (−2.4, 0.3)	77	12.0 ± 5.6	11.6 ± 7.4	−0.3 (−1.7, 1.0)	0.7 (−1.1, 2.4)	0.4427
PVR (mL)		78	26.7 ± 21.9	30.9 ± 35.0	3.3 (−3.1, 9.8)	77	31.3 ± 26.6	37.1 ± 35.4	6.8 (0.3, 13.4)	3.5 (−5.1, 12.0)	0.4253

*Note:* Values at week 0 and week 12 are presented as mean ± SD or *n* (%), while changes are expressed as point estimates with 95% confidence intervals. On the seven‐point scale of the PGI, a score of 1–2 was considered very much satisfied and a score of 1–3 was considered satisfied.

Abbreviations: HUS, hours to undisturbed sleep; MCIC, minimal clinically important change; PGI, patient global impression; PVR, post‐void residual urine volume; Qmax, maximum urinary flow rate; SD, standard deviation.

The proportion of patients who achieved the OABSS MCIC was 66.7% in the vibegron add‐on group and 47.4% in the α_1_‐blocker monotherapy group. The between‐group difference was 19.3% (95% CI: 3.8 to 34.8), which was statistically significant (*p* = 0.0214).

In the PGI assessment at week 12, 55.8% and 88.3% of patients in the vibegron add‐on therapy group reported being “very much satisfied” and “satisfied”, respectively, compared with 16.9% and 57.1% in the *α*_1_‐blocker monotherapy group, respectively. The values from both assessments were significantly higher in the vibegron add‐on therapy group than in the *α*_1_‐blocker monotherapy group (Table 2).

Using Qmax and PVR, the changes in Qmax from baseline (week 0) to week 12 were −1.0 mL/s and −0.3 mL/s in the *α*_1_‐blocker monotherapy and vibegron add‐on therapy groups, respectively, while the corresponding changes in PVR were 3.3 mL and 6.8 mL, respectively, with no statistically significant differences between groups (Table 2).

**FIGURE 3:**
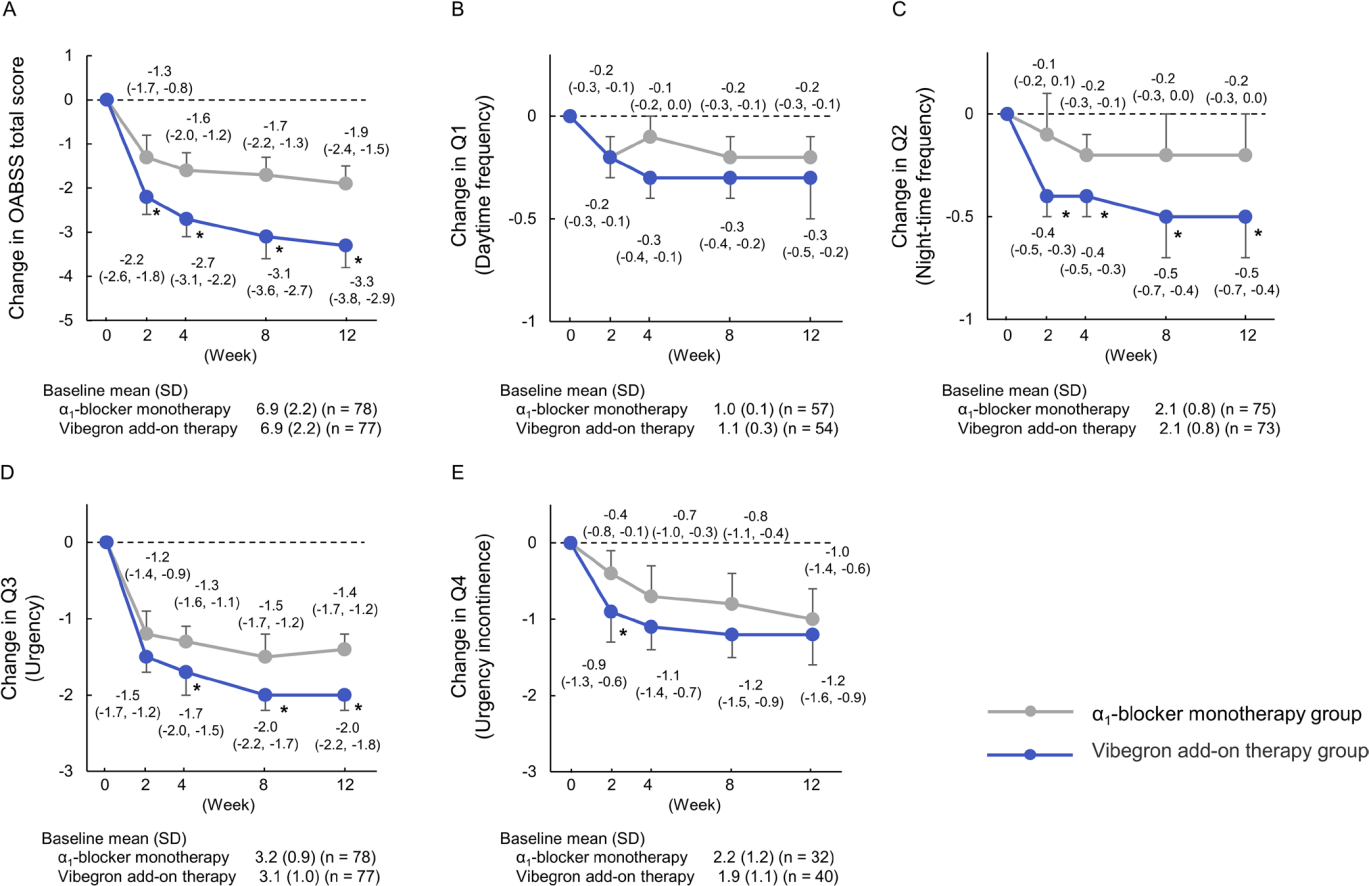
Longitudinal changes in total and subscale OABSS from baseline to week 12. A: OABSS total score; B: Q1. daytime frequency; C: Q2. night‐time frequency; D: Q3. Urgency; E: Q4. urgency incontinence. Data points, bars and values indicate least‐squares mean (95% CI) of the changes from baseline; an asterisk (*) indicates a between‐group difference with *p* < 0.05. CI, confidence interval; LS, least squares; OABSS, overactive bladder symptom score; SD, standard deviation.

**FIGURE 4:**
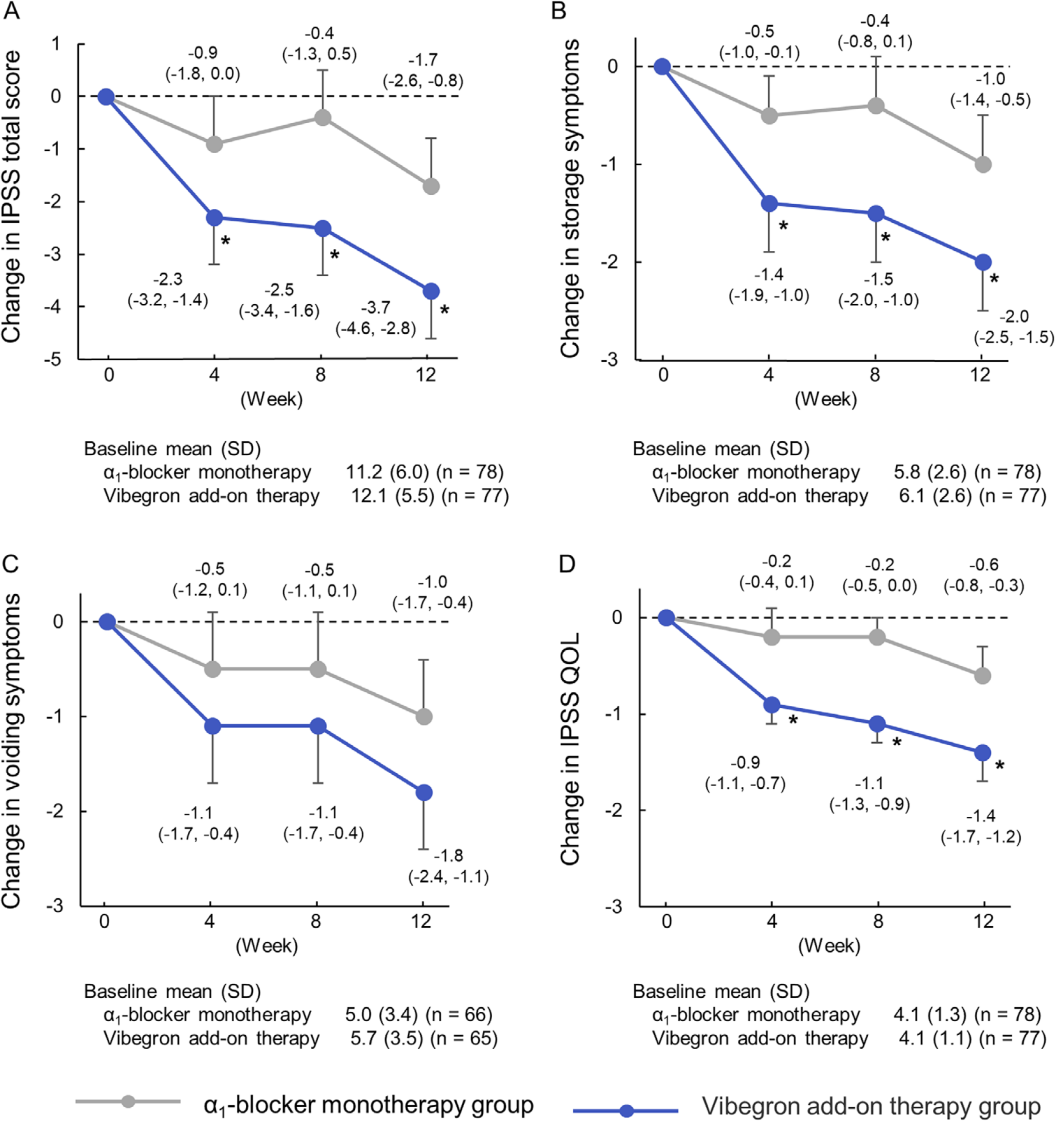
Longitudinal changes in IPSS (total, storage, voiding) and IPSS‐QOL from baseline to week 12. A: IPSS total score; B: Storage symptoms; C: Voiding symptoms; D: IPSS‐QOL. Data points, bars and values indicate least‐squares mean (95% CI) of the changes from baseline, with an asterisk (*) indicating that the difference between groups was *p* < 0.05. CI, confidence interval; IPSS, international prostate symptom score; LS, least squares; QOL, quality of life; SD, standard deviation.

In analyses of change from baseline (week 0) to each evaluation time point, the vibegron add‐on therapy group demonstrated significantly greater reductions than did the *α*_1_‐blocker monotherapy group in the OABSS total score and Q2 from week 2 through week 12 (Figure 3). In IPSS‐based assessments, significant decreases in the IPSS total score, IPSS‐QOL score, and the storage‐symptom score were observed at all evaluated time points (Figure 4).

### 3.3 Subgroup Analyses

The efficacy of vibegron as an add‐on therapy in various patient subgroups is presented in Table S2. Regarding the OABSS total score, vibegron exhibited significant improvement from baseline to week 12 compared with *α*_1_‐blocker monotherapy in the following subgroups: age < 75 years, prostate volume < 50 mL and ≥ 50 mL, OAB duration < 7 and ≥ 7 months, complications (no and yes), and OABSS total score < 8 and ≥ 8. Vibegron maintained similar efficacy trends regardless of the baseline *α*_1_‐adrenergic antagonist used, whether silodosin, tamsulosin, or naftopidil.

### 3.4 Safety Endpoints

All TEAEs are summarized in Table 3. TEAEs were observed in 12.7% (10 cases) and 22.8% (18 cases) of the *α*_1_‐blocker monotherapy and vibegron add‐on therapy groups, respectively. Drug‐related TEAEs were observed in 6.3% (five cases) of the vibegron add‐on therapy group, including one, three, and one case of constipation, increased residual urine volume, and dysuria, respectively. Serious TEAEs comprised coronavirus disease 2019 and cardiac valve disease in the vibegron add‐on therapy group. No serious drug‐related TEAEs were observed.

**TABLE 3:** Number and percentage of patients with treatment‐emergent adverse events (safety analysis set).

Names in AE: SOC (bold) and PT (plain)	*α*_1_‐blocker monotherapy group (*n* = 79) *n* (%)	Vibegron add‐on therapy group (*n* = 79) *n* (%)
All TEAEs	10 (12.7)	18 (22.8)
Drug‐related TEAEs	0 (0.0)	5 (6.3)
Serious TEAEs	0 (0.0)	2 (2.5)
Drug‐related serious TEAEs	0 (0.0)	0 (0.0)
All TEAEs in any treatment group		
Infections and Infestations	3 (3.8)	7 (8.9)
Dermatophytosis of nail		1 (1.3)
Nasopharyngitis	3 (3.8)	4 (5.1)
COVID‐19		2 (2.5)
Metabolism and nutrition disorders	1 (1.3)	0 (0.0)
Hyperlipidaemia	1 (1.3)	
Nervous system disorders	1 (1.3)	0 (0.0)
Normal pressure hydrocephalus	1 (1.3)	
Cardiac disorders	0 (0.0)	1 (1.3)
Cardiac valve disease		1 (1.3)
Respiratory, thoracic and mediastinal disorders	1 (1.3)	0 (0.0)
Cough	1 (1.3)	
Gastrointestinal disorders	0 (0.0)	4 (5.1)
Abdominal pain		1 (1.3)
Constipation		1 (1.3)
Diarrhea		1 (1.3)
Enterocolitis		1 (1.3)
Skin and subcutaneous tissue disorders	1 (1.3)	2 (2.5)
Blister		1 (1.3)
Eczema		1 (1.3)
Stasis dermatitis	1 (1.3)	
Musculoskeletal and connective tissue disorders	2 (2.5)	1 (1.3)
Arthralgia		1 (1.3)
Back pain	1 (1.3)	
Osteoarthritis	1 (1.3)	
Renal and urinary disorders	1 (1.3)	2 (2.5)
Dysuria		2 (2.5)
Haematuria	1 (1.3)	
Investigations	0 (0.0)	3 (3.8)
Residual urine volume increased		3 (3.8)
Injury, poisoning and procedural complications	1 (1.3)	1 (1.3)
Fall		1 (1.3)
Heat illness	1 (1.3)	

*Note:* Blank space shows 0 (0).

Abbreviations: PT, preferred terms; SOC, System organ class; TEAEs, treatment‐emergent adverse events.

## 4 Discussion

To our knowledge, this study represents the first prospective investigation to demonstrate the efficacy of vibegron 50 mg once daily in an RCT for patients with BPH‐associated OAB who experience persistent OAB symptoms despite *α*_1_‐blocker therapy. Compared to the *α*_1_‐blocker monotherapy group, the vibegron add‐on therapy group demonstrated superior efficacy in the primary endpoint of change in OABSS total score, as well as in secondary endpoints including various parameters in the bladder diary, OABSS, IPSS score, IPSS‐QOL, and PGI assessment. Furthermore, regarding safety evaluation, no serious drug‐related TEAEs were observed in the vibegron add‐on treatment group.

Although the vibegron Phase 3 trial conducted in Japan demonstrated the efficacy and safety of a 50 mg dose, the study population had several limitations: males comprised only 9.7% of participants, the mean age was relatively young at 58.0 years, and *α*_1_‐blockers were prohibited as concomitant medications [9]. While the efficacy of combining *α*_1_‐blockers with vibegron 75 mg has been reported [17], real‐world data regarding the combination of vibegron 50 mg with *α*_1_‐blockers, which serve as foundational therapy for BPH treatment, remain lacking. Here, vibegron 50 mg add‐on therapy exhibited superior therapeutic outcomes compared to *α*_1_‐blocker monotherapy.

In the vibegron add‐on therapy group, therapeutic efficacy, as assessed by the primary endpoint OABSS total score, became evident as early as week 2, with significant improvements compared with the *α*_1_‐blocker monotherapy group, which were maintained through week 12. Comparable improvements were observed in the OABSS Q2 score, indicating that vibegron alleviated nocturia symptoms at week 2. In evaluations of *β*_3_‐adrenergic agonists for comparable conditions, mirabegron studies have generally set assessment time points at or after week 4, and early treatment effects have not been examined. In this study, we demonstrate for the first time that vibegron 50 mg provides an early benefit for persistent OAB symptoms in men with BPH who remain symptomatic despite *α*_1_‐blocker therapy. In OAB treatment, low medication persistence remains a significant concern [18]. Moreover, early treatment benefit perception has been suggested to be associated with higher treatment satisfaction and improved treatment persistence [19]. Therefore, the early effect onset demonstrated in the present study may represent clinically meaningful real‐world management information.

Here, between‐group differences in urgency urinary incontinence episode outcomes were not statistically significant. Only a small proportion of participants exhibited these symptoms (16 patients with urgency urinary incontinence in both groups), which may have limited the statistical power to detect differences. In randomized clinical trials comparing mirabegron, a *β*_3_‐adrenergic agonist, with placebo in male patients with OAB receiving *α*_1_‐blockers, significant effects on urgency urinary incontinence likewise have not been demonstrated [6]. Future studies that enroll sufficient numbers of patients with these symptoms are warranted to evaluate the potential benefit of vibegron add‐on therapy.

Regarding nocturia, the number of nocturia episodes was significantly reduced in the vibegron add‐on therapy group at 12 weeks, accompanied by a significant HUS extension. In research evaluating mirabegron in patients with comparable conditions, no significant effects on nocturia frequency have been reported, whereas the *β*_3_‐adrenergic agonist vibegron yielded different results in the present setting. Notably, nocturia has been identified as the most bothersome lower urinary tract symptom [2], and reductions in nocturnal voiding episodes and extension of HUS may contribute to improved treatment satisfaction. Given concerns about the impact of nocturia on mortality and fracture rates [20], the efficacy of vibegron add‐on therapy demonstrated in this study may have favorable effects in appropriate patients, potentially leading to improved patient prognosis.

In subgroup analyses using the primary endpoint (change in OABSS total score), the analysis restricted to patients aged ≥ 75 years suggested a trend toward benefit of vibegron add‐on therapy compared with continued *α*_1_‐blocker monotherapy (between‐group difference, **−**0.7 [95% CI: −1.4 to 0.0]); however, the difference was not statistically significant (*p* = 0.0642). The pathophysiology and clinical expression of BPH‐related OAB are multifactorial and complex, and prior research on mirabegron suggests that longer symptom duration may negatively influence clinical responsiveness [21]. Therefore, rigorous evaluation of vibegron in patients aged ≥ 75 years may require a sample size and design calibrated to the anticipated effect size. Separately, in analyses stratified by the background *α*_1_‐blocker, the addition of vibegron demonstrated benefit with silodosin, tamsulosin, and naftopidil, suggesting the utility of vibegron for persistent OAB symptoms despite *α*_1_‐blocker therapy.

TEAEs did not show characteristic increases with vibegron administration. Nonetheless, drug‐related TEAEs occurred in five cases in the vibegron add‐on therapy group, including constipation, increased residual urine volume, and dysuria. These voiding function‐related adverse events were not acute urinary retention and did not require catheterization. Vibegron has been demonstrated pharmacologically not to increase PVR [22], and urinary function‐related adverse events were uncommon in the phase 3 study conducted in Japan [9]. Nevertheless, because most BPH patients in real‐world clinical practice are older adults, with a high proportion who may have compromised detrusor function [23], and considering that *α*_1_‐blocker effectiveness may differ according to prostatic pathological conditions [24]. As noted in a previous commentary [25], when initiating vibegron add‐on therapy, careful consideration of age, concomitant medications (including anticholinergics) that may affect voiding function, and baseline post‐void residual volume is advisable. Careful follow‐up with monitoring of voiding status and PVR is also recommended.

This study has some limitations. First, although randomized, it was an open‐label trial; therefore, placebo (expectancy) effects cannot be fully excluded. To clearly establish the difference between the vibegron add‐on therapy group and the *α*_1_‐blocker monotherapy group, a placebo‐controlled comparative study would be necessary. Nevertheless, vibegron add‐on therapy may be clearly more beneficial for improving OAB symptoms than simply continuing *α*_1_‐blocker monotherapy. Second, although PDE5 inhibitors represent another therapeutic option for BPH in addition to *α*_1_‐blockers, data on combining PDE5 inhibitors with vibegron are not available from this study.

## 5 Conclusion

This study evaluated the efficacy and safety of vibegron add‐on therapy in patients with BPH with persistent OAB symptoms despite continuous *α*_1_‐blocker treatment. Vibegron demonstrated significant improvement in persistent OAB symptoms compared with *α*_1_‐blocker monotherapy, achieving superior patient treatment satisfaction outcomes. Vibegron was well‐tolerated, and no major safety concerns related to voiding function were identified. Once‐daily vibegron 50 mg add‐on therapy with *α*_1_‐blockers may be an efficacious treatment option for BPH treatment. The study findings contribute to expanding evidence‐based management of lower urinary tract dysfunction.

## Author Contributions

M.Y. had full access to all the data in the study and takes responsibility for the integrity of the data and the accuracy of the data analysis. M.Y. and N.H. contributed to the conception and design of the study. All authors drafted and/or critically revised the manuscript and approved the final version and agreed to its submission.

## Funding

This work was supported by Kyorin Pharmaceutical Co. Ltd. and Kissei Pharmaceutical Co. Ltd.

## Conflicts of Interest

This work was supported by research funding from Kyorin Pharmaceutical Co. Ltd. and Kissei Pharmaceutical Co. Ltd. M.Y. has received personal fees (including lecture and consulting fees) from Astellas, Kyorin, Zeria, Taiho, and JURO Science. H.K. has received personal fees from Kyorin. Y.N. has received personal fees from MSD, AstraZeneca, and Astellas. N.H. has received research funding from Pfizer and Johnson & Johnson; personal fees from Kissei, Kyorin, and Astellas, and has served on an advisory board for Kissei. M.I. is a full‐time employee of Kyorin Pharmaceutical Co. Ltd. S.K. is a full‐time employee of Kissei Pharmaceutical Co. Ltd.

## Supporting information


**Figure S1:** Study design (schematic).

**Table S1:** Inclusion and exclusion criteria.
**Table S2:** Subgroup analysis of change in OABSS total score from baseline to week 12.

## Data Availability

Research data are not shared.
