# Supplementary figures and images for: Efficacy and Safety of Vibegron Add‐On Therapy for Persistent Overactive Bladder Symptoms in Benign Prostatic Hyperplasia Patients With α1‐Blocker Treatment: A Multi‐Center Prospective Randomized Controlled Study (VATON Study)
Source: Low Urin Tract Symptoms. 2026 Mar 13;18(2):e70053. doi: 10.1111/luts.70053 (PMC12988341; doi:10.1111/luts.70053)

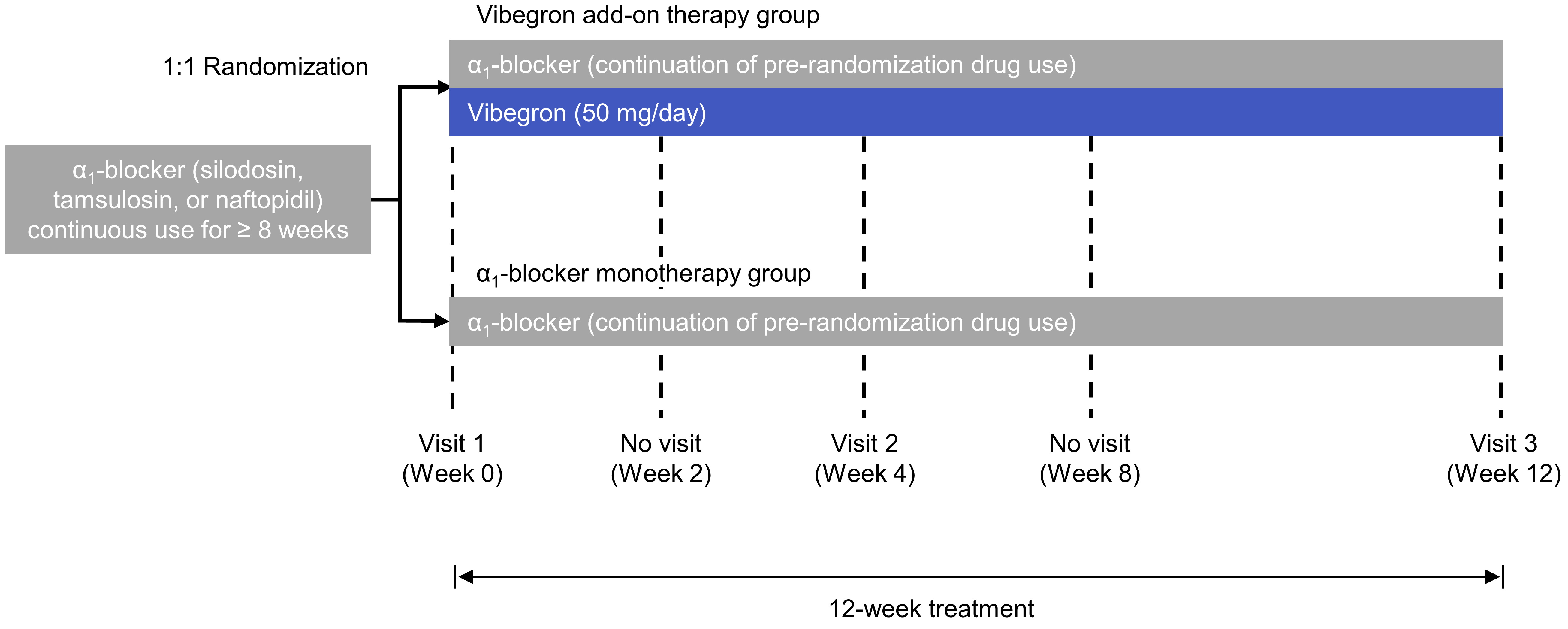

Supplement: Supplementary file 1 — Figure S1: Study design (schematic). [file LUTS-18-e70053-s001.tif]
