# Supplementary material for: Efficacy and Safety of Vibegron Add‐On Therapy for Persistent Overactive Bladder Symptoms in Benign Prostatic Hyperplasia Patients With α1‐Blocker Treatment: A Multi‐Center Prospective Randomized Controlled Study (VATON Study)
Source: Low Urin Tract Symptoms. 2026 Mar 13;18(2):e70053. doi: 10.1111/luts.70053 (PMC12988341; doi:10.1111/luts.70053)
Supplement: Supplementary file 2 — Table S1: Inclusion and exclusion criteria. Table S2: Subgroup analysis of change in OABSS total score from baseline to week 12. [file LUTS-18-e70053-s002.docx]

**Supplementary Figure legend and Table Captions**

Supplementary Figure 1. Study design (schematic)

Supplementary Table 1. Inclusion and exclusion criteria.

| **Criteria** | **Confirmation time point** | |  |
| --- | --- | --- | --- |
| Inclusion criteria | At the time of obtaining consent | Visit 1 (0 weeks) | |
| - 1. Patients aged ≥50 years, male patients | ○ | − | |
| 1. Patients diagnosed with BPH through basic evaluation and other diagnostic methods | ○ | − | |
| 1. Patients who agree to comply with the criteria for using concomitant medications/therapies, including any restricted medications | ○ | − | |
| 1. Patients who have been using an α_1_-blocker (silodosin, tamsulosin, or naftopidil) at the same dosage for ≥8 weeks before Visit 1 | ○* | ○ | |
| 1. Patients with a prostate volume ≥20 mL (either transabdominal or transrectal ultrasound within 6 months) | − | ○ | |
| 1. Patients with an OABSS Q3 score of ≥ 2 points and an OABSS total score of ≥ 3 points | − | ○ | |
| 1. Patients who are able to properly record their bladder diaries | − | ○ | |
| 1. Patients who provide written consent | ○ | − | |
| Exclusion criteria |  |  | |
| 1. Patients with neurogenic bladder (physician's discretion) | ○ | − | |
| 1. Patients with a history of BPH surgery | ○ | − | |
| 1. Patients with urinary retention | ○ | − | |
| 1. Patients with stress urinary incontinence only | ○ | − | |
| 1. Patients with orthostatic hypotension | ○ | − | |
| 1. Patients with a prolonged indwelling catheter or self-administration of urine | ○ | − | |
| 1. Patients with complications that present with symptoms similar to those of OAB (urinary retention, urinary lithiasis, urinary tract infection, interstitial cystitis, and prostatitis) that, in the opinion of the investigator or subinvestigator, may affect the evaluation of this study | ○ | − | |
| 1. Patients with a history of injury, surgery, or neurodegenerative disease (e.g., multiple sclerosis) affecting the lower urinary tract and innervation | ○ | − | |
| 1. Patients with bladder or prostate cancer who are undergoing or will undergo treatment | ○ | − | |
| 1. Patients with hypersensitivity to vibegron | ○ | − | |
| 1. Patients with serious cardiac disease or severe hepatic dysfunction | ○ | − | |
| 1. Patients with residual urine volume ≥ 100 mL | − | ○^†^ | |
| 1. Patients with a confirmed maximum urine flow rate (Qmax) value of <5 mL/s (when a single voiding volume of >100 mL is measured) | − | ○^†^ | |
| 1. Patients with polyuria (total daily voiding volume averaging ≥3000 mL) | − | ○ | |
| 1. Patients who cannot comply with the concomitant use of prohibited drugs or therapies for a specified period of time | − | ○ | |
| 1. Patients who have taken new or changed the dosage or administration of concomitantly restricted drugs during the specified period. | − | ○ | |
| 1. Patients who the investigator or subinvestigator judges to be inappropriate to participate in this study (e.g., patients diagnosed with dementia) | ○ | − | |

* For a patient who has not been using the same α_1_-blocker for 8 weeks at the time of consent, confirm that the patient can use the same α_1_-blocker at the same dosage and administration method for 8 weeks until the scheduled date of Visit 1.

† If the previous residual urine volume or urine flow test results within 4 weeks before Visit 1 are used, the decision should be based on those results.

OABSS, overactive bladder symptom score; BPH, benign prostatic hyperplasia

Supplementary Table 2. Subgroup analysis of change in OABSS total score from baseline to week 12.

|  | | **α_1_-blocker monotherapy group** | | | | **Vibegron add-on therapy group** | | | | **Difference in changes between groups** | |
| --- | --- | --- | --- | --- | --- | --- | --- | --- | --- | --- | --- |
| Subgroup | Category | n | Week 0 | Week 12 | Change (95% CI) | n | Week 0 | Week 12 | Change  (95% CI) | Difference  (95% CI) | p value |
| Age (year) | < 75 | 34 | 7.0 ± 2.4 | 5.4 ± 3.0 | -1.5  (-2.2, -0.9) | 32 | 6.8 ± 2.4 | 3.0 ± 1.9 | -3.9  (-4.6, -3.2) | -2.3  (-3.2, -1.4) | < 0.0001 |
|  | ≥ 75 | 44 | 6.8 ± 2.0 | 4.5 ± 2.4 | -2.2  (-2.8, -1.7) | 45 | 7.0 ± 2.1 | 4.0 ± 2.5 | -2.9  (-3.5, -2.4) | -0.7  (-1.4, 0.0) | 0.0642 |
| Prostate volume (mL) | < 50 | 69 | 6.9 ± 2.2 | 4.8 ± 2.7 | -2.0  (-2.5, -1.6) | 64 | 6.8 ± 2.2 | 3.5 ± 2.3 | -3.2  (-3.7, -2.7) | -1.2  (-1.8, -0.5) | 0.0003 |
|  | ≥ 50 | 9 | 6.4 ± 2.1 | 5.6 ± 2.9 | -1.0  (-2.3, 0.4) | 13 | 7.8 ± 2.1 | 3.8 ± 2.4 | -3.9  (-4.9, -2.8) | -2.9  (-4.5, -1.3) | 0.0005 |
| Duration of OAB (mo) | < 7 | 45 | 6.9 ± 2.3 | 4.7 ± 2.7 | -2.3  (-2.9, -1.7) | 31 | 7.3 ± 2.4 | 3.0 ± 1.6 | -4.1  (-4.8, -3.4) | -1.9  (-2.8, -1.0) | < 0.0001 |
|  | ≥ 7 | 33 | 6.9 ± 1.9 | 5.3 ± 2.7 | -1.5  (-2.1, -0.9) | 46 | 6.7 ± 2.1 | 4.0 ± 2.6 | -2.8 (-3.3, -2.2) | -1.3 (-2.0, -0.5) | 0.001 |
| Complications | No | 18 | 7.0 ± 1.9 | 5.5 ± 2.6 | -1.5 (-2.4, -0.6) | 25 | 7.2 ± 2.1 | 3.2 ± 1.8 | -4.0 (-4.8, -3.2) | -2.5 (-3.6, -1.3) | < 0.0001 |
|  | Yes | 60 | 6.8 ± 2.2 | 4.8 ± 2.7 | -2.0 (-2.5, -1.6) | 52 | 6.8 ± 2.3 | 3.8 ± 2.5 | -3.0 (-3.5, -2.5) | -1.0 (-1.6, -0.3) | 0.0047 |
| OABSS total score | < 8 | 50 | 5.7 ± 1.3 | 4.3 ± 2.3 | -1.4  (-1.8, -0.9) | 49 | 5.6 ± 1.2 | 3.0 ± 1.7 | -2.6  (-3.1, -2.2) | -1.2  (-1.8, -0.6) | < 0.0001 |
|  | ≥ 8 | 26 | 9.2 ± 1.6 | 6.2 ± 3.0 | -2.9  (-3.8, -2.1) | 26 | 9.4 ± 1.7 | 4.7 ± 2.8 | -4.7  (-5.6, -3.9) | -1.8  (-2.9, -0.7) | 0.0015 |
| α_1_-blocker | Silodosin | 23 | 7.3 ±2.4 | 5.1 ±2.8 | -2.0  (-2.9, -1.2) | 23 | 6.9 ±2.1 | 3.5 ±2.1 | -3.5  (-4.3, -2.7) | -1.5  (-2.6, -0.3) | 0.0111 |
|  | Tamsulosin | 26 | 6.9 ±2.3 | 4.7 ±2.6 | -2.3  (-3.0, -1.5) | 23 | 7.2 ±2.0 | 3.2 ±1.8 | -4.0  (-4.8, -3.2) | -1.7  (-2.8, -0.7) | 0.0014 |
|  | Naftopidil | 29 | 6.5 ±1.9 | 5.0 ±2.7 | -1.5  (-2.2, -0.8) | 31 | 6.7 ±2.5 | 3.9 ±2.7 | -2.7  (-3.3, -2.1) | -1.2  (-2.1, -0.3) | 0.0083 |

Values at weeks 0 and 12 are presented as mean ± SD, while changes are expressed as point estimates with 95% CIs. Abbreviations: CI, confidence interval; OABSS, overactive bladder symptom score; SD, standard deviation.
